# Immunotherapy for Dogs: Still Running Behind Humans

**DOI:** 10.3389/fimmu.2021.665784

**Published:** 2021-08-05

**Authors:** Hans Klingemann

**Affiliations:** ImmunityBio Inc., Culver, CA, United States

**Keywords:** dogs, immunotherapy, canine, T-cells, natural killer cells, monoclonal antibodies

## Abstract

Despite all good intentions, dogs are still running behind humans in effective cancer immunotherapies. The more effective treatments in humans, like infusions of CAR-T and NK-cells are not broadly pursued for canines due to significant costs, the rather complicated logistics and the lack of targetable surface antigens. Monoclonal antibodies are challenging to develop considering the limited knowledge about canine target antigens and about their mode of action. Although immunogenic vaccines could be less costly, this approach is hampered by the fact that cancer by itself is immuno-suppressive and any preceding chemotherapy may suppress any clinically meaningful immune response. This review – rather than providing a comprehensive listing of all available immunotherapies for dogs, aims at pointing out the issues that are holding back this field but which hopefully can be addressed so that dogs can “catch up” with what is available to humans.

## Introduction

Back in 2017, I published a review on the immunotherapy options for dogs with cancer. Somewhat provocatively the paper was entitled: *Immunotherapy for dogs: Running behind humans* (1). The years have gone by and it is time to assess if our dogs have caught up. Looking at what has been accomplished in those 4 years, it is fair to conclude that canine immunotherapy has come a few steps closer but certainly a lot more work needs to be done.

There are several reasons why this area is moving rather slowly (**Table 1**). Any immunotherapy that profit-minded companies would be interested in commercializing for the canine market would have to be offered at a prize that dog owners feel comfortable paying. Since only about 10% of dog owners have health insurance for their pet, there is a ceiling what owners can and will spend on complex treatments. Especially cell-based cancer treatments (T-cells, NK-cells) require good manufacturing practices (GMP) compliant *ex vivo* cell expansion, special instrumentation, cytokines and trained personnel, easily accumulating costs of several thousands of dollars. Hence, companies (with some exceptions) will not be eager to develop those treatments.

**Table 1:** Reasons why immunotherapy for dogs is still lagging behind humans.

Developing cellular immunotherapies are expensive and pharmaceutical companies have limited enthusiasm to develop them for canines assuming the projected profit is limited Targetable surface molecules (of the CD family) are less well characterized than in humans Effector mechanism for monoclonal antibodies are incompletely known: which cells can mediate ADCC and which IgG subclasses are utilized for ADCC Lack of reagents to characterize comprehensively dog’s immune cells and cytokine profile Conducting randomized controlled clinical trials is challenging (breed variability, disease staging, compliance in case of progression)

To potentially alleviate this issue, “comparative oncology” has gained some popularity [reviewed in (2)]. The premise is that there are overlapping similarities/characteristics of certain cancers between humans and dogs that are also reflected at the genetic level. Hence hard to fund academic investigators may welcome this model, which is supported by NIH grants, as any positive result could potentially benefit drug development for humans. However, the concern is that the dog is becoming a better mouse model for exploring new therapeutics for humans. Also, the *comparative oncology* concept might only apply to cancers that are common in dogs and humans like lymphoma, osteosarcoma and melanoma, but certainly not for the majority of human malignancies such as cancer of the lung, colon, breast, prostate and pancreas. Although the human and the canine genome have roughly 80% homology, discovery of new immuno-therapeutics based on this homology is limited and largely restricted to small molecules. A recent study tested the cross-reactivity of seven FDA approved human immune checkpoint inhibitors (ICI) with dog immune cells and only two IPIs had (limited) cross-reactivity (S. Pantelyushin, personal communication). Moreover, If given to dogs, an immune response would be expected to those human proteins. Hence there are limitations what comparative oncology can offer when it comes to testing *human* cell and protein based therapeutics for applications in dogs.

The following review is not about a comprehensive listing of immunotherapies that are available for dogs, being developed or may even be promising – there are excellent reviews that have accomplished just that (3–5). This article is more of an assessment of what the issues are that are holding dogs back from catching up with immunotherapy options that are available for humans.

## Cellular Immunotherapy With Cytotoxic Lymphocytes (T – Cells and NK -Cells)

The most effective cellular immunotherapies in the *human* space as of now - besides allogeneic stem cell transplantation - involve *CAR engineered T-cells (CAR-T)* and *monoclonal antibodies (mAb)* (6). The costs for preparing a CAR T-cell product for a human patient is currently in the $ 350K range. Even with the smaller size of a dog and somewhat less stringent GMP and regulatory issues to comply with, the costs for this treatment will still be substantial – but cost considerations are just one aspect: it still is unknown how best to generate CAR-T cells that can target canine cancer antigens – which are also insufficiently characterized. The dog equivalent of CD19 has not been identified yet and only one study looked at anti-CD20 CAR-T cells transfected with mRNA (7). As expected for mRNA transfection, the duration of CAR expression was short. It is known from human CAR-T cell studies that the T-cells have to clonally expand and persist in the recipient to show efficacy (8). Based on this short expression, no conclusions can be drawn about potential efficacy and side effects with CAR-T cells in canine patients.

At the recent SITC (Society for Immunotherapy of Cancer) meeting, the Seattle group presented data on CAR-T cell treatment in 2 dogs (9). The CAR-T cells were directed against the human B7/H3 surface antigen on solid tumors which the authors showed to be cross-reactive between humans and canines. In contrast to the previous study, the T-cells were transduced retrovirally which resulted in about 70% transduction efficiency. The canine recipients also received lymphodepleting chemotherapy (Fludarabine/Cyclophosphamide) prior to infusion. Persistence of the CAR-T cells in the canine blood could be demonstrated for several weeks *via* a co-transfected EGFR tag. 10^9^ CAR-T cells/m^2^ were infused and only mild liver enzyme abnormalities were noted.

Some preliminary *in vitro* results were recently reported with canine -T cells transfected with RNA for a caninenized CAR construct. This construct targeted the canine IL13a2 receptor through the human Fab portion of the CAR (the remaining CAR components were canine) (10). The target tumor for this approach would be glioma, but the issue of time limited RNA expression and any immunogenicity of the human Fab need to be addressed.

Considering the challenges with CAR-T cell therapy in dogs, alternative cellular therapies deserve consideration. ELIAS Animal Health has developed a cellular therapy for osteosarcoma that uses a vaccination step with the patient’s tumor tissue followed by apheresis to harvest the dog’s T – cells. Those are then *ex vivo* activated (using a proprietary formula) and expanded. The cell preparation is sent back to the veterinarian for infusion (11). An update on the study results were recently presented (Bryan, 2020, personal communication)^1^, and if the data hold, this could present a treatment options for dogs with (non-metastatic) osteosarcoma and potentially for other cancers as well. A larger controlled study with carboplatinum as the control group is underway.

*NK cells* are currently seeing a surge in clinical trials in humans. Their advantage over T- cells is their safety profile (no cytokine storm when expressing a CAR) but their life span is shorter. For the human patient, investigators are using cord blood or peripheral blood NK cells which may require depletion of any allogeneic T-cells (12). The continuously growing NK-92 cell line (13) provides a logistically easier effector NK-cell pool and clinical trials with PD-L1 expressing NK-92 cells (t-haNK™) in humans are ongoing (14). With the advent of CRISPR/Cas9 those cells can be genetically engineered such that they are no longer immunogeneic for canine T-cells. Treatment of canine osteosarcoma and histiocytoma with cells from the human clonal NK/T cell line (TALL 104) in the late 1990’s had shown some encouraging anti-tumor effects (15, 16).

Human NK cells express the characteristic CD56 antigen and are negative for CD3 expression. In contrast, NK cells from dogs are less well defined (17). Since their first description in 2000 (18), not much additional characteristics have been identified. What has been described in the literature is that that these “NK-like” cells have granular morphology, express CD5 at low density and are CD3 and CD56 negative (19, 20). The canine surface markers coded by the NCR1 gene p46 (21) and CD94 (22) seem to be expressed variably on these cells and their full relevance is still poorly understood.

This lack of deeper knowledge about canine NK cells has negatively affected the clinical development of NK-based immunotherapy for dogs. The team at UC Davies has infused *ex vivo* activated “NK-like” cells in conjunction with local radiation for treatment of osteosarcoma (23). The canine NK cells were isolated from PBMC based on CD5dim/NKp46 expression and then expanded *ex vivo* for two weeks on a K562 feeder layer modified with mIL21 and in the presence of recombinant human IL-2 (100 IU/mL). Those expanded NK cells were then infused after local tumor radiation. The rationale for this approach is based on their pre-clinical work showing that radiation sensitizes the tumor to the cytotoxic effects of NK cells and also directs their migration. The potential synergy between local radiation and immunotherapy has been reviewed by Thamm (24).

In humans, NK cells are also essential cytotoxic effector cells for antibody dependent cellular cytotoxicity (ADCC) by binding mAbs through the CD16 Fcγ-receptor. For dogs it has not been conclusively demonstrated that “NK-like” cells can mediate ADCC against antibody-coated tumor cells and that they express a human Fcγ-receptor/CD16 equivalent.

## Cytokines

Although a portfolio of canine recombinant cytokines is available for laboratory research, no canine specific cytokines are commercially available for *treatment* of dogs (4). Human IL-2 and IL-12 (including NHS-IL-12, a fully human IgG immunocytokine) have been given to dogs with cancer – but these cytokines generally require higher doses than in humans. Except for a modest efficacy signal with NHS-IL-12 (25), convincing data on *in vivo* tumor control are largely missing. The same is true for the intra-tumor electro-application of a plasmid based IL-12 construct (26, 27).

Under the auspices of the PRECINCT Canine Trials Network the (human) IL-15 superagonist Anktiva^R^ is being tested in combination with inhaled (human) IL-15 in dogs with melanoma or osteosarcoma driven lung metastases [PIs: Canter RJ, Rebhun RB. [https://www.precinctnetwork.org].

## Monoclonal Antibodies

A striking example about the gap between what is available to humans *versus* dogs is the area of mAbs. There are no mAbs for dogs approved and effective for cancer therapy. The CD20 antibody from Arantana/Elanco is approved by the USDA (28) but has not stood the test of time for effectiveness (29).

Not unexpectedly, human mAbs generally do not cross-react with canine target cells – in the case of rituximab, the most widely used mAb in the treatment of human lymphoma, this can be traced back to a single amino acid difference in the active binding site (30). There is some suggestion that cetuximab (anti-EGFR) and trastuzumab (anti-Her-2) can bind to some canine cancer cell lines (31). The larger issue relates to the fact that the human protein will induce an anti-human immune response in dogs (humoral and cellular). Some caninenized mAbs (only the complementary sequences are non-canine) have received conditional approval by the USDA for lymphoma (i.e. Blontress^®^, Tactress^®^). Disappointingly no peer-reviewed clinical evidence of efficacy for those mAbs has been published. Elanco’s anti-CD20 mAb (1E4) has shown to deplete B-cells in healthy Beagles with an *in vivo* half-life of about 2 weeks, but the mAb is still undergoing clinical efficacy testing (32). Recently a chimeric (rat) anti-canine CD20 mAb (defucosylated) has been reported (33) which significantly depleted B-cells in the blood of beagles (n=8) lasting for about 4 weeks. Clinical data are still pending. The Pittsburgh group recently described a anti-canine CD19 mAb (4E9) based on immunohistology. Data to its therapeutic relevance are not available yet (34).

For mAb treatment to be effective in dogs, it is important to remember how mAbs deploy their therapeutic effect: (i) neutralizing antibodies bind to the target cells and prevent those cells from further functioning/dividing; (ii) direct cytotoxic mechanisms involve complement mediated cell lysis (CMC) and (iii) antibody dependent cellular cytotoxicity (ADCC). Not much is known about the complement system in dogs. ADCC in humans is executed predominantly by natural killer (NK) cells through their Fc-receptor (CD16) that binds mAbs of the IgG1 or IgG3 subclass. A CD16 like receptor has not been described on canine NK cells and little is also known about which IgG subtypes in dogs can execute ADCC. The IgG subclasses in dogs are designated A,B,C,D and it appears from preliminary studies that B and D may be involved in ADCC (35). Since NK cells are rather incompletely defined in dogs, their contribution in ADCC is largely unknown although an earlier study had suggested some ADCC mediated by canine large granular lymphocytes isolated based on surface markers (CD3− CD21− CD5− TCRαβ− TCRγδ), activated and expanded for about two weeks with various cytokines on a K562 feeder layer (36).

Sine only a rather limited number of *canine* cancer antigens are known, investigators have been looking into *human* mAbs that may be cross-reactive. The clinical relevance though is limited considering that those antibodies, like trastuzumab or cetuximab, mostly work through ADCC (37, 38). In addition, the immunogenicity of these human proteins is a concern.

Stipulated by the positive outcome data with immune checkpoint inhibitors (ICIs) in some human cancers, especially melanoma and lymphoma (39), the search for canine mAb equivalents has gained some traction (40, 41). First reports about the distribution and function especially of PD-1 for immune-cells and its ligand PD-L1 on tumor tissues are just emerging (42, 43). A canine chimeric mAb (the Fab site being rat-derived) against PD-L1 was tested in dogs with oral melanoma and sarcoma (44). All nine tumors (n=7 melanoma, n=2 sarcoma) expressed PD-L1, based on immunohistology. Only two temporary partial responses after multiple infusions were seen in a dog with melanoma and in one dog with sarcoma.

A Japanese team recently presented data on two canine *PD-1* antibodies: one being a rat-dog chimeric mAb and the other one is a caninenized antibody with the complementary segment being rat derived (45). Thirty dogs with oral melanoma were treated with a mean of about 8 infusions. Side effects (mostly grade 1) were seen in 19/30 dogs but with one death caused by the mAb. Four dogs with stage IV oral melanoma had an objective temporary response and some stable diseases were noted with the majority of dogs progressing on treatment. Compared to matched historical controls, overall survival was not prolonged.

Since human and canine immune checkpoint inhibitors (ICIs) have about 70-80% sequence homology, a Swiss group tested the blocking potential and functional effects of seven FDA-approved human ICIs, targeting CTLA-4 and the PD-1/PD-L1 against canine tissue (S. Pantelyushin, personal communication). Out of seven candidates only *atezolizumab* and to a lesser extent *avelumab* increased canine T-cell cytokine production *in vitro*. Although these *in vitro* data are interesting, they will unlikely stipulate trials in dogs because of the immunogenic potential of the human protein structure. Considering the available data with canine ICI, it would be reasonable to conclude that the relevance of current ICIs in dogs is rather limited especially when taking the cost/benefit ratio into account.

## Tumor Vaccines and Oncolytic Viruses

If proven effective, tumor vaccines could be logistically simple and provide a relative affordable immunotherapy for dogs. Different approaches are currently being considered. The vaccine is usually administered after initial treatment of the cancer when the patient is in presumed remission. Vaccines can be generated against neoepitopes on cancer cells, often against driver oncogens like mutated p53, KRAS, Her-2 applied as peptides (46). Recent data presented at the 2020 SITC meeting (47) showed in a mouse model that *autologous* tumor infiltrating lymphocytes (TIL) were only effective against p53 expressing tumors after the T-cells had been engineered to express an anti-p53 specific T-cell receptor. This observation is relevant with regard to a more general problem with tumor vaccination: unmodified autologous T-cells are ineffective effector cells, having become tolerant and “exhausted” due to the underlying cancer and chemotherapy.

Another group of cancer vaccines is supposed to stimulate the dog’s immune system non-specifically without providing a cancer specific antigen. There are currently two DNA based vaccines approved by the USDA: *Oncept^®^* (human DNA based) is given to dogs with oral melanoma. Unfortunately, no properly controlled studies beyond comparison with historical controls are available (48, 49). Essentially the same applies for a murine DNA vaccine (also from Merial/Boehringer) for canine lymphoma. Both vaccines are widely used in the veterinary practice – their reasonable costs, the fact that they are USDA approved and that the veterinarian feels that something can be offered to the dog’s cancer treatment, are motivators for prescribing these vaccines.

A recombinant xenogeneic (human Her-2/neu expressing) *Listeria* based vaccine against osteosarcoma was reported in 2016 from the University of Pittsburgh (50). Eighteen dogs with appendicular osteosarcoma following amputation and chemotherapy received the vaccine. The median survival time of the treatment group appeared to be significantly longer than the historical controls. Unfortunately, no “Listeria only” treatment group was included in that study and no immune response parameters against the human Her-2 sequence were reported. Adverse events in that study were mild to moderate and primarily consisted of fever, lethargy, and nausea/vomiting. In 2017 the *Comparative Oncology Trials Consortium* at the NCI initiated a larger trial in 80 dogs to test the vaccine across various centers. The study recently closed and reporting of results is pending. However, outcome data may be inconsequential as some serious Listeria-related infections were reported in 4 dogs that were treated with the preparation outside of the study (51, 52). Elanco Inc. (Greenfield, IN) which had been granted conditional licensure from the USDA, has decided not to develop the vaccine further.

Oncolytic viruses are believed to initiate a similar immunotherapy response as vaccines in addition to providing a potential direct cytolytic effect (53). Oncolytic viruses can have selectivity for malignant cells where they replicate and - by lysing the “host” cell – expose and present the antigens to immune cells. Only limited research in this area has been done for canine cancers although some initial positive results are available in canine melanoma with an adenovirus based (human) CD40 Ligand (54).

The Vaccination Against Canine Cancer Study (VACCS) is in a special category as the vaccine is a “gemisch” of approximately 30 abnormal proteins found on the surface of common canine cancers (lymphoma, osteosarcoma, hemangiosarcoma and mastocytoma) (55). These “abnormal” proteins are the result of frameshift mutations resulting in improperly coded RNA. The vaccine is given prophylactically to dogs at high risk while they are still healthy. It remains to be seen if this approach can generate memory T-cells that are able to react when a tumor arises that expresses the protein that was part of the initial vaccine.

## Immune-Active Small Molecule Candidates

Genetic abnormalities of canine cancers have become the emphasis for small molecule drug development to potentially interfere with the abnormality. It remains to be seen if sequence variations in the genome are sufficiently informative. Human studies suggest that genetic changes in cancer are multiple and complex and that it is more relevant to look at proteomics as not all genetic abnormalities result in a tumor inducing/promoting molecule (56) Efforts are underway to apply transcriptional analysis of tumor tissue to come up with immune-active combinations that would target any alteration. As part of the Cancer Moonshot U01 grant, clinical studies are underway using such targets as: CXCR1, CCR2, VGFR/Flt3/gp130 (S. Dow, personal communication).

## Immunological Modulation of the Tumor Microenvironment

Increasing attention is focused around the relevance of the tumor microenvironment (TME) to support and maintain malignant transformation. Our knowledge about the immunological make-up of the TME is still quite sketchy largely due to the lack of reliable *in vitro* models. From studies with human cells it is known that T-regulatory cells (Tregs) and immunosuppressive (type II) macrophages in the TME can negatively affect the anti-tumor immune response (57). It was recently reported that blockade of CCR4 signaling with a humanized antibodies could deplete Tregs in a canine model of invasive bladder cancer resulting in sustained tumor regression and prolonged survival (58)

## What Next?

In order to offer dogs novel immunotherapies that truly benefits them, we need more basic research that defines in more detail the components of the canine immune system and its interaction with the tumor in the context of the its microenvironment. Also, a more detailed analysis of cancer antigens, their immunogenicity and targetability need to occur. Just initiating clinical trials in dogs to explore new drugs/molecules that potentially would benefit humans without comprehensive knowledge of the specifics of the dog’s immune system, will not benefit them.

## Author Contributions

The author confirms being the sole contributor of this work and has approved it for publication.

## Conflict of Interest

The author is currently Chief Science Officer- Cellular at ImmunityBio. He is also co-founder of and equity holder in the company.

## Publisher’s Note

All claims expressed in this article are solely those of the authors and do not necessarily represent those of their affiliated organizations, or those of the publisher, the editors and the reviewers. Any product that may be evaluated in this article, or claim that may be made by its manufacturer, is not guaranteed or endorsed by the publisher.

## Footnotes

1 Paws for Cure Research Symposium
